# Interdisciplinary Confusion and Resolution in the Context of Moral Machines

**DOI:** 10.1007/s11948-022-00378-1

**Published:** 2022-05-19

**Authors:** Jakob Stenseke

**Affiliations:** Department of Philosophy, Lund University, Lund, Sweden

**Keywords:** Machine ethics, AI ethics, Artificial moral agent, Interdisciplinarity, Disciplinary perspectives, Applied epistemology

## Abstract

Recent advancements in artificial intelligence (AI) have fueled widespread academic discourse on the ethics of AI within and across a diverse set of disciplines. One notable subfield of AI ethics is *machine ethics*, which seeks to implement ethical considerations into AI systems. However, since different research efforts within machine ethics have discipline-specific concepts, practices, and goals, the resulting body of work is pestered with conflict and confusion as opposed to fruitful synergies. The aim of this paper is to explore ways to alleviate these issues, both on a practical and theoretical level of analysis. First, we describe two approaches to machine ethics: the *philosophical approach* and the *engineering approach* and show how tensions between the two arise due to discipline specific practices and aims. Using the concept of *disciplinary capture*, we then discuss potential promises and pitfalls to cross-disciplinary collaboration. Drawing on recent work in philosophy of science, we finally describe how *metacognitive scaffolds* can be used to avoid epistemological obstacles and foster innovative collaboration in AI ethics in general and machine ethics in particular.

## Introduction

*Grand challenges* are complex problems of global concern that call for inter- and transdisciplinary research efforts (Brooks et al., 2009). As intelligent artificial systems continue to enter and transform more aspects of human life, artificial intelligence (AI) arguably poses several grand challenges; from ensuring that research, development, and application of AI adheres to ethical and societal considerations in the short-term, to the prevention of long-term catastrophic risks of future AI. Consequentially, the last decade has yielded a boom of research tackling the ethics of AI from the perspective of social science, philosophy, engineering, and law.

*Machine ethics* (ME) is a subfield of AI ethics that seeks to endow artificial systems, software and hardware alike, with ethical faculties (Wallach & Allen, 2008). Today—with more self-driving cars occupying public roads, and a wide variety of robots used as assistants and companions in education, care, and beyond—it is increasingly hard to deny the need for “ethical AI”, i.e., artificial systems with some form of ethical considerations implemented into their design. After all, if AI were to carry on its current trajectory of replacing conventional human occupations, e.g., teachers, drivers, doctors, and even soldiers, one might also expect that they will meet the ethical standards usually presupposed by those roles. Accordingly, the emerging field of ME have attained a growing interest among researchers, with a diverse body of work that spans from theoretical discourses on what artificial moral agents (AMAs) are (Moor, 2011), whether AMAs are possible or desirable (Behdadi & Munthe, 2020), to technical and experimentally oriented work on prototypical AMAs (Tolmeijer et al., 2020).

However, since different branches within ME have discipline-specific concepts, practices, and goals, the field is caught up in conceptual confusion and profound disagreements. In particular, there is a large gap between, on the one hand, conceptual and normative work on artificial morality driven by moral philosophy, and on the other hand, technical and experimental work driven by computer science. Unfortunately, the gap between ethics and technology is by no means exclusive to ME. For instance, while many initiatives in AI ethics have more or less converged on a set of guidelines and principles (Floridi & Cowls, 2019), their ability to have any major impact on the ethical development of AI has been heavily criticized (Hagendorff, 2020; Mittelstadt, 2019). Instead, without the appropriate mechanisms to impose their own normative claims, AI guidelines might merely act as “ethics washing” strategies for private companies and public institutions. The same gap has obscured the prospects of moral machines, with philosophers speculating about moral consequences of machines that cannot be built, and computer scientists reducing complex moral domains to optimization problems that are in turn ‘solved’ by simplifications of human-like moral abilities. While previous surveys in ME have helped to clarify and classify technical approaches to moral machines,^1^ no work has exposed the foundations that underpin the multitude of perspectives that pervade in the field at large, and the potential sources of conflict they give rise to. In turn, instead of promoting productive collaborations that utilize the strengths of various disciplines, such divisions can serve to cement incommensurable visions and perspectives of the near- and long-term challenges of AI.

The main goal of this paper is to explore practical and theoretical ways to resolve some of the aforementioned issues and foster inter- and transdisciplinary research in ME. First, we characterize two main branches of ME and show how tensions between the two arise due to discipline-specific practices and aims. We then discuss potential promises and pitfalls to cross-disciplinary collaboration. Drawing on recent work in philosophy of science (Baalen & Boon, 2019), we then describe how *metacognitive scaffolds* can be used to clarify the diverging epistemologies and research goals that underpin conflicting views on machine morality. In particular, we discuss elements of a disciplinary matrix that can help to resolve interdisciplinary confusion by explicating crucial but not always salient commitments of discipline-specific research efforts.

## The Philosophy and Engineering of Moral Machines

The majority of work in ME is approached from two disciplinary families, namely moral philosophy and computer science (Tolmeijer et al., 2020). Both families have their own distinct history, practices, methods, and goals. Although it is difficult to identify a method common to all work in moral philosophy, the central aim of the field is to—in more or less systematic was—resolve questions about what is “good” and “bad”; whether it is to determine what is moral in particular cases (applied ethics), advance general standards of what is moral (normative ethics), or explore the meaning and nature of morality (metaethics). Computer science—and the interrelated fields of computer engineering, information systems, software engineering, and AI^2^—lies in the intersection of *mathematics* (computers as physical realizations of mathematical entities (Hoare, 1969, 1993)), *engineering* (constructing computational artefacts, or “the engineering of mathematics” (Hartmanis, 1981)), and *empirical science* (Newell & Simon, 2007). The primary focus is to explore what is possible to do with computational systems, analyzed by “all analytical and measurement means available” (Newell & Simon, 2007, p. 114).^3^ Generally speaking, while moral philosophy seeks to resolve questions about what humans *ought* to do, computer science seeks to understand what computer systems *can* do. In turn, this division allows us to describe two main types of ME:

1. *The philosophical approach to machine ethics* (PME)—the conceptual exploration of what computer systems *ought* to do, and correspondingly, what systems *ought* to be built.
2. *The engineering approach to machine ethics* (EME)—the exploration of what kind of morality *can* be implemented in computer systems, and what moral systems *can* be built.

Of course, not all approaches to moral machines can be rightfully characterized as either PME or EME, as the field encompass all kinds of combinations of the two.^4^ There is, however, a significant divergence between projects that explore what kind of moral considerations one can implement in a computational system (EME), and work that reflect upon, justify, or condemn a particular kind of machine morality (PME). A major source of divergence resides in the fact that, while PME is not necessarily constrained to what is (at least currently) technically possible, EME is not necessarily constrained by the ethical considerations posed by the former. This gives rise to a rich and diverse landscape of approaches to moral machines, including technical and experimental work on AMAs,^5^ conceptual work on more or less feasible AMAs (Bauer, 2020; Howard & Muntean, 2017), work on moral cognition expected of morally competent AMAs (Malle & Scheutz, 2020), discussions on whether and to what extent AMAs *can* have a moral agency or status (Malle, 2016; Sparrow, 2021), debates on whether and to what extent AI *should* be implemented with morality,^6^ normative work based on possible future AI (Bostrom, 2017; Metzinger, 2021; Tonkens, 2012), and efforts to ensure safe and explainable reliable AI that aligns with human values (Amodei et al., 2016; Gabriel, 2020; Gunning et al., 2019). The disparity has consequently spawned a great number of conflicting visions of moral machines, ranging from the most optimistic to the most pessimistic accounts, some justified on the basis of current technical feasibility, while others are based on mere long-term possibility.^7^

After all, ethics and artificial intelligence are both multifaceted and complex phenomena that cannot, on any level of analysis, be reduced to simple elements that would allow for a straight-forward integration. From naïve optimism about future AI to Neo-Luddite technophobia, substantial disagreement is not only expected, but perhaps necessary to encompass a heterogeneity of diverse approaches to moral machines. But even if one might embrace this diversity, it also gives rise to debates and oppositions that are, in the words of Behdadi and Munthe (2020), “conceptually confused and practically inert” (Behdadi & Munthe, 2020, p. 195).^8^ While we do not claim that all conflicts can or even should be resolved, we do believe that a significant amount can be disentangled by clarifying the epistemologies, practices, and goals that underpin different approaches. In the following two sections we will take a closer look at solutions that ideally can pave the way for cross-disciplinary integration and collaboration.

## Interdisciplinary Collaboration and Disciplinary Capture

One seemingly straightforward way to reconcile PME and EME is to work together in interdisciplinary research efforts, and in this section, we will discuss promises and potential pitfalls for such endeavors in the context of ME.

Against the background of the earlier made characterization, for joint PME and EME research efforts, a first step can be to recognize the diverging constraints of moral *oughts* and technical *cans*; that is, to reach a mutual understanding of the moral constraints posed by the former and the technical constraints of the latter. While PME researchers can conceive of both moral excellence and immoral maleficence of future AI, it might carry little normative power to EME researchers if it is not grounded in technical feasibility. Conversely, lacking the philosophical competence, EME researchers might develop and implement moral machines in various real-life domains without any rigorous justification of why their machine is in fact needed or desired. Furthermore, supposedly moral machines of the EME perspective might not even be considered worthy of the epithet ‘moral’ in the perspective of PME, since they fail to satisfy essential criteria of moral agency as it has been construed within the tradition of moral philosophy.^9^

We thus argue that the most promising collaborations mutually utilize the constraints and possibilities of both perspectives. What kind of morality a machine *ought* to have should be informed by what it *can* feasibly have, and vice versa, provided that a certain machine *can* be built should be guided by moral considerations of whether it *should* be.^10^ Productive collaborations are also those that effectively make use of the disciplinary-specific advantages of the two branches, in particular the imaginative and critical elements of philosophical inquiries, and the formal and empirical tools of computer science. The constructive and deconstructive power of PME can for instance be illustrated in the fact that it offers ways to justify the construction of a certain moral machine, e.g., on the basis of some moral and societal goods, but also, on similar grounds, argue for a global moratorium on the research and development of certain machines.^11^ Likewise, besides providing the means to de facto develop and construct machines, a major advantage of EME is the possibility to analyze computational models by a wide range of analytical means, such as mathematical proofs of correctness, statistical reliability, and software simulations. Ultimately, a successful integration of PME and EME would in turn guide the development of moral machines that are not just technically feasible, but ethically justified, and grounded on rigorous philosophical inquiry of moral concepts in a computational context.

However, there are a number of possible pitfalls to the kind of ideal integration just described, of which some are common to other forms of interdisciplinary work. As discussed by Brister (2016), both more overt and less overt epistemic disagreements about facts, causes, research goals, and evidentiary standards can result in *disciplinary capture*, meaning that the standards, values, and methodological presumptions of one discipline take precedence over another.^12^ In order to avoid disciplinary capture, it is therefore relevant to identify how it can occur in interdisciplinary collaborations within ME.

Disciplinary capture by EME can more generally be viewed as part of the common trend where data, mathematical models, and computational tools are increasingly used to assist or even transform entire research areas.^13^ More particularly, if the joint research effort is dominated by EME, there is potential risk for what can be described as *computational simplification,* meaning that complex phenomena—e.g., moral behavior, moral cognition, moral values, or moral environments—are simplified and reduced to elements that can be formalized, quantified, and executed in computational models. A typical example is the concept of ‘rational agent’—as it is conventionally construed in economics, game theory and AI—which reduces complex human behavior to self-interested agents that seeks to maximize some given utility (Russell & Norvig, 2002). Another example is the use of quantifiable metrics, as it potentially fails to account for qualitatively and holistically construed values and perspectives (Duffy & Chenail, 2009).^14^ Yet another example is the tendency to reduce complex domains to optimization problems that can be solved by maximizing or minimizing a specified objective function. Consequentially, there is a risk that research efforts dominated by EME replace equivocal and ‘rich’ moral values, concepts, and theories with simplified ones in order to produce functional applications in well-defined computational settings, without any regard for how such values, concepts, and theories are situated within the history of human self-understanding, nor how they are related to the broader landscape of moral behavior and cognition. To that end, it is no coincidence that deontology and consequentialism are the two normative frameworks that are most widely used for technical implementations in machine ethics. In their survey of implementations in machine ethics, Tolmeijer et al. (2020) found that 28 out of 50 surveyed implementations are based on either deontology, consequentialism, or a combination of the two. While deontology conveniently corresponds to conditional statements that drives software programming (e.g., “If X → do Y”), consequentialism’s emphasis on quantifiable utility elegantly resonates with reward-functions of reinforcement learning and objective functions in mathematical optimization.

A related and potentially more potent source of disciplinary capture by EME stems from the goals of knowledge production inherent to computer science research. In particular, the epistemic goal of much work in computer science—and correspondingly, the methods used to reach those goals—is to define computational artefacts and conduct experiments on them (Tucker, 2004). In joint research efforts driven by EME, it is therefore expected that the main research result consists of a computational system along with experiments that shows what it can do, as opposed to, e.g., a critical reflection of what it ought to do. The disciplinary “construction as knowledge” ethos also makes EME more susceptible to the influence of market interests. Indeed, R&D of AI within and outside academia is to a large extent driven by market interests that fund and support the construction of systems that can be turned into economic profit.^15^ For EME in particular, this includes the industry prospects of self-driving vehicles and social care robots. The epistemic goals and methods of computational simplification of EME can also be mutually reinforcing; phenomenon needs to be simplified in order to be formalized and computed, and computed in order to satisfy the epistemic aims of computer science. In effect, the work of PME researchers within EME dominated collaborations might only serve as a form of “ethics washing”,^16^ e.g., by merely providing an ethical reflection on some possible consequences of the constructed artefact, but leaving out a justification of why the same artefact in fact should be created in the first place.

Disciplinary capture can also occur in the opposite direction. As opposed to computational simplification, a PME dominated collaboration might instead pave the way for what can be described as “conceptual obfuscation” or “moral gatekeeping”. The first refers to the use of intangible concepts that, in the view of EME, resists formal definition and thus cannot be ‘compiled’ into executable machine commands. For instance, a PME researcher might draw from a rich background of philosophical resources in order to construe a certain concept that is supposedly essential for human morality, e.g., consciousness or autonomy. However, since the language used to construe the moral concepts cannot be translated into a computational context (let alone a neuroscientific one), the EME drowns in an ocean of semantic confusion. While philosophy can allow for a certain interpretative headroom (e.g., due to the use of ambiguous and rich terms stemming from various traditions of human self-understanding) and disagreement (e.g., in the sense that there is usually no general consensus in philosophical debates^17^), the formal language necessary for AI development does not.^18^ In turn, PME dominated research can potentially result in “moral gatekeeping”, e.g., by arguing that a machine cannot be moral because it lacks a certain moral aspect X, and that X, for various reasons, cannot be computed (Sparrow, 2021).

Due to different epistemic aims, a research effort captured by PME might also result in a collaboration that fails to effectively utilize the competence of EME. For instance, the research goal might be to produce a critical perspective on machine morality aimed towards engineers. However, due to the use of concepts that only makes sense in a philosophical context, it fails to engage its target audience. Furthermore, while EME is driven to produce computational artifacts, critical PME is propelled by generating normative conclusions, which in turn carries the potential to influence policy makers. A PME-led project could thus provide a condemnatory view on the prospects of moral machines without any regard of the de facto technical dimensions of AI development, which, in the worst case could lead to unjustified political moratoriums.

However, simply identifying how some forms of disciplinary capture can occur within ME is not sufficient to prevent it from occurring.^19^ After all, computer science and moral philosophy are highly specialized disciplines that require vast and different kinds of cognitive skills. In the next section we will describe how metacognitive knowledge, represented as *metacognitive scaffolds*, can be used to develop the skills required to further promote and execute interdisciplinary research.

## Disciplinary Matrices as Metacognitive Scaffolds

Intuitively, certain metacognitive skills are needed to integrate the special competences of two or more academic disciplines. However, based on studies in the educational literature, the teaching of such skills remains underdeveloped in higher education (MacLeod, 2018; Thorén & Persson, 2013). As a possible solution, Baalen and Boon (2019) have proposed the use of *metacognitive scaffolds* as an epistemic tool that can be used to articulate and analyze how a certain discipline generates and applies knowledge. The reason is that researchers more or less unknowingly adopt a certain *disciplinary perspective*, i.e., a set of disciplinary-specific beliefs, methods, and values that enables and constrains how they conduct research. Importantly, a disciplinary perspective can become ‘second nature’ for researchers, in the sense that “experts are hardly aware of how the specificities of their disciplinary contribute to the ways in which they do their research and generate epistemic results” (Baalen & Boon, 2019, p. 16). Following Kuhn’s idea of disciplinary matrices (Kuhn, 1970), Baalen and Boon argue that the elements of a disciplinary perspective can be characterized in terms of a *disciplinary matrix*, which explicates the relevant epistemic elements associated with a certain perspective (Baalen & Boon, 2019).^20^ The disciplinary matrix can in turn be used as a metacognitive scaffold to articulate disciplinary perspectives, effectively providing a way to foster communication and resolve epistemic conflicts in interdisciplinary research projects.

In the same vein, we propose ten topics that can serve to elucidate disciplinary perspectives relevant to the field of ME, namely *consciousness*, *autonomy*, *rationality*, *normative ethics, metaethics*, *implementation*, *technology*, *research aim, justification*, and *technological assessment* (summarized in Table 1)*.* While the list of suggested topics is by no means exhaustive, we believe it can provide an important starting-point for inter- and transdisciplinary projects in ME to better analyze and understand their respective views and commitments.

**Table 1:** List of topics (left column) with possible questions and answers (right column) that can be used to describe, analyze, and compare views central for different approaches to machine morality (inspired by Baalen and Boon (2019))

Consciousness	Q: What sort of consciousness is sufficient/necessary for morality? A: Dualism, physicalism, functionalism, computationalism, behaviorism
Autonomy	Q: What kind of autonomy is sufficient/necessary for moral agency and responsibility? A: Self-legislative (Kantian), independent from human supervision (AI)
Rationality	Q: What sort of rationality is sufficient/necessary for morality? A: Instrumental (rational agent), human-like rationality, Hobbesian empiricism, Kantian rationalism
Normative ethics	Q: What is morally good? A: Maximization of well-being (utilitarianism), duties and rights (deontology), virtues and flourishing (virtue ethics)
Metaethics	Q1: What is the nature of moral judgements? A: universalism, relativism, nihilism Q2: What is the meaning of moral terms? A: cognitivism, non-cognitivism Q3: Is moral knowledge possible?A: empiricism, rationalism, intuitionism, skepticism Q4: What is the nature of ethics? A: philosophical, social, psychological, biological Q5: How is morality evaluated? A: societal good, human experts, moral law
Implementation	Q: How should ethics be implemented in machines? A: Top-down, bottom-up, hybrid
Technology	Q: What are the most suitable technical methods for developing moral machines? A: Logical reasoning, probability, machine learning, optimization
Research aim	Q: What is the overall aim of the research? A: Epistemic, normative, critical, theoretical, practical, constructive, monetary
Justification	Q: How is the research justified? A: Inevitability, harm-prevention, public trust, preventing immoral use, moral superiority of AMAs, better understanding of morality
Technological assessment	Q: How realistic is the explored artificial moral agent? A: Theoretically possible in the long-term, practically feasible with current technology

The first three topics—consciousness, autonomy, and rationality—all carry enormous weight in the Western philosophical tradition, and as a consequence, they are decisive for particular views on machine morality; e.g., whether and to what extent machines *can* or *should* be moral. The first row exemplifies philosophical views on consciousness that are central to machine ethics.^21^ For instance, Champagne and Tonkens (2015); Coeckelbergh (2010); Himma (2009); Johansson (2010); Purves et al. (2015); Sparrow (2007) all argue that the capacity for phenomenal consciousness is central for moral agency.^22^ By contrast, authors such as Anderson (2008); Floridi and Sanders (2004); Gerdes and Øhrstrøm (2015); Veruggio et al. (2016), have rejected the necessity of phenomenal consciousness for moral agency on the more pragmatic epistemic basis that it remains difficult to ascribe consciousness to others from a third-person perspective (e.g., a neuroscientific or computational point of view).

Autonomy and free will—following the Kantian tradition (Kant, 2008) or the “Principle of Alternative Possibilities” (Frankfurt, 1969)—are, in a similar way, often advocated as necessary requirements for moral agency, dignity, and responsibility (Friedman & Kahn Jr, 1992; Hellström, 2013; Himma, 2009).^23^ However, human-centered conceptions of autonomy differ significantly from the functionally defined notions of autonomy used in AI development, where it often refers to an ability to perform a certain task independent from human supervision or control (Mostafa et al., 2019).^24^

Rationality plays a similar key role in discussions about machine morality and moral competence (Coeckelbergh, 2009; Davis, 2012; Himma, 2009). Although no one denies the central importance rationality has for morality, the term is pestered with semantic obfuscation in the sense that it is frequently influenced by disciplinary perspectives and more or less salient assumptions about human rationality, often in intricate conjunction with conceptions about phenomenal consciousness and autonomy. This includes “maximizing self-interest of rational agents” in game theory and economics (Coleman & Fararo, 1992), “goal-directed behavior” in AI development (Russell & Norvig, 2002), “following reason” (e.g., having reasons for actions and beliefs), understanding intentions and desires of others (Dennett, 1989), higher-order cognitive abilities for rational inquiry and conscious deliberation (e.g., Aristotle’s *animale rationale*), “empathic rationality” capable of moral imagination and reflective equilibrium (Purves et al., 2015), Humean empiricism (“reason is the slave of passions”), and Kantian rationality (according to the law of the autonomous will).

Essentially, there are major conceptual gaps between, on the one hand, notions of rationality, autonomy, and consciousness that have been central to philosophical explanation and human self-understanding, and on the other hand, similar terms that are reimagined and modelled within modern AI development. It is therefore crucial to acknowledge the role conceptualizations play in disciplinary perspectives of PME and EME. If one’s research aim is to construct an (allegedly) ethical machine, one would necessarily start from the assumption that it is in fact possible to do so. As a result, one might commit to computationalism about cognition and properties necessary for morality; not because it is the most compelling theory, but because it works in favor of one’s epistemic aim. Furthermore, what seems like trivial premises for some disciplines, might be conceived as disrespectful or even harmful for others; e.g., ignoring the results of millennia-old debates, failing to engage at a normative or societal level, or disregarding what is technically feasible, scientifically explainable, or empirically supported.

The fourth topic serves to elucidate views on normative ethics that divides most approaches in machine ethics.^25^ Note that this does not only include questions regarding “what is good” as such, but also how to *do* good (e.g., moral actions and decisions that *are* good in themselves or *lead* to good outcomes) or *be* good (e.g., in terms of a moral character). There is also an important difference between ethical theory as *normative ideal* and in terms of *action-guidance* (Erman & Möller, 2013). As a complement, the fifth topic serves to explicate metaethical views regarding the ontological, semantic, and epistemological commitments of moral practices, and how these in turn relate to normative theory. It can also be used to articulate views beyond the conventional debates in metaethics, e.g.., stressing the social (norms, community, culture), psychological (dispositions, emotions, attitudes), or biological nature of ethics (e.g., evolution of cooperation and altruism). Importantly, it should also address how morality is evaluated—e.g., by human experts, moral law, utility, social good, cooperation among self-interested agents—as it profoundly influences one’s approach to machine ethics.^26^

Topic six and seven are based on two dimensions that divides technical approaches to moral machines, namely how morality should best be implemented and technically realized. More specifically, the former asks—following a scheme proposed by (Allen et al., 2005)—whether moral behavior should be implemented in a 'top-down’ fashion (e.g. based on pre-determined principles and knowledge), learned through a ‘bottom-up’ process, or in a combination of both.^27^ The latter, in turn, serves to analyze the computational methods that are most suitably used to realize the implementation, which might include logical reasoning (Bringsjord & Taylor, 2012), Bayesian techniques (Cloos, 2005), or machine learning (Stenseke, 2021).^28^

The eight topic serves to clarify the aim and purpose of the research, e.g., whether it is to conceptually explore a certain kind of AMA, contribute to the desirability or feasibility debates on moral agency, to create an AI system based on a particular normative theory, or to criticize a certain approach to machine ethics. More importantly, although it is conventional that the aim of a contribution is stated in the work itself (e.g., as a research aim or objective), it is often influenced by broader and less salient outlooks and assumptions stemming from one’s disciplinary perspective, e.g., about what AI is and what it should be.

In a similar vein, the ninth topic offers an opportunity to justify the research project, e.g., provide reasons *why* AMAs are desirable or useful and for *whom*. For instance, Van Wynsberghe and Robbins (2019) have critically examined six reasons machine ethicists offer in favor for the development of AMAs: inevitability (the emergence of AMAs are bound to happen by necessity), prevention of harm (AMAs should be developed so as to prevent machines from hurting humans), public trust (AMAs would help to increase the public trust of AI systems), preventing immoral use (AMAs will prevent humans from misusing robots), moral superiority (AMAs have the potential of being morally superior to humans), or to understand morality (developing AMAs will lead to a better understanding of human morality). They conclude that none of the provided reasons withstand critical scrutiny nor work in practice, and consequentially, they urge machine ethicists to give better reasons and think more carefully about why we need to develop moral machines in the first place.

Finally, the last topic offers room to clarify *technological assessment*, i.e., whether the discussed AI system is practically feasible or only theoretically possible in short-, mid-, or long-term. While the primary purpose of the technological assessment is to settle confusion between the speculative and realistic—e.g., is the research based on AI technology of today, or does it explore some possible AI of the long-term future?—it can also be used to explicate one’s view on epistemic uncertainty in relation to potentially catastrophic risks of future AI.^29^

By addressing these topics, we hope that researchers within PME and EME can get a better understanding of how ‘knowledge’—of epistemological views, aims, methods, and justifications—is created, and more importantly: how different disciplines do this in different ways. As such, the topics can serve as metacognitive scaffolds to analyze and reconstruct ‘knowledge’ in a way that enables interdisciplinary collaborations to thrive.

## Conclusion

We have explored the gap between ethics and technology by focusing on the conflict between discipline-specific approaches to machine ethics. Importantly, we have shown how work in machine ethics are propelled and shaped by the elements of disciplinary perspectives—e.g., epistemic and normative aims, values, and methods—that lead to conflicting views on the prospect of machine morality as well as confusion. We have argued that such divisions might foster incommensurable perspectives on machine morality, which in turn curtails what disciplinary-specific approaches could meaningfully contribute to the overarching challenges of the field. Instead, to produce research relevant for the entire field, ethicists and engineers should think carefully about how their work could be strengthened and enriched by perspectives beyond their own discipline. Of course, not all conflicts can be resolved by simply working together, nor by explicating the epistemological and normative underpinnings of one’s research. There are also benefits with heterogeneity and disagreement in the sense that disciplinary plurality can account for a wide variety of values, methods, and visions that cannot—at least not easily—be integrated into a unified whole. Nonetheless, based on our work, we believe that at least some disputes and misunderstandings can be unraveled in a way that is helpful for engineering, philosophical, and interdisciplinary approaches to machine ethics. Furthermore, while this paper has focused on issues in machine ethics, we hope that similar work can assist in resolving tensions and disciplinary disarray within AI ethics at large.

In summary, this paper supports three claims:

1. To meet the grand challenges posed by AI, disciplinary perspectives need to be further integrated.
2. In the field of machine ethics, integration can be achieved through interdisciplinary collaboration between moral philosophy and computer science, in particular by utilizing the moral *oughts* posed by the former and the technical *cans* of the latter.
3. Interdisciplinary research within ME can be further promoted by (i) identifying and avoiding disciplinary capture; and (ii) articulating the underlying views that supports conflicting perspectives on machine morality (e.g., with the help of metacognitive scaffolds).

## Footnotes

1 See Allen et al. (2005), Cervantes et al. (2020), Moor (2011), Tolmeijer et al. (2020).

2 The difficulty of classifying the broad and ever-growing research landscape of computer science has ironically been tackled by computational means. According to a recent taxonomy, which was automatically generated from a data set of roughly 16 million publications, computer science can be divided into 14,164 topics with 162,121 semantic relationships (Salatino et al., 2020). By contrast, the manually handled ACM Computing Classification System contains about 2000 topics (Rous, 2012).

3 See also Parnas (1985) for a critical account of how computer science has transformed in the age of software engineering.

4 In fact, what we in this work characterize as PME can in some cases be viewed as the philosophical critique of EME. Furthermore, it is possible to distinguish two types of PME; normative PME that criticizes or advocates a particular approach to machine morality, and speculative PME that conceptually explores some possible moral machine without necessary drawing normative conclusions.

5 For two recent surveys on implementations in machine ethics, see Cervantes et al. (2020) and Tolmeijer et al. (2020).

6 For a detailed discussion on both the feasibility and desirability of AMAs, see Behdadi and Munthe (2020), Bryson (2010), Himma (2009), Johnson and Miller (2008), Sharkey (2017), Tonkens (2009), Van Wynsberghe and Robbins (2019), Yampolskiy (2013).

7 This includes idealistic views on the moral superiority of future machines (Arkin, 2007; Dietrich, 2001; Gips, 1994), apocalypticism about the existential risks posed by future super-intelligence (Bostrom, 2017), cautious conservatism in the face of artificial suffering by means of synthetic phenomenology (Metzinger, 2021), and calls for moderation based on the current state of AI (Cervantes et al., 2020; Farisco et al., 2020), to only name a few.

8 In their analysis of the AMA debate, Behdadi and Munthe (2020) finds that confusion about central concepts—e.g., rationality, consciousness, free will, and autonomy—makes it “unclear which positions are incompatible and the extent to which opponents in the debate are even addressing the same thing” (p. 195). Furthermore, they argue that conceptual discussions on moral agency are of limited practical use for more stressing problems, e.g., of *whether* and to *what extent* AI systems could be meaningfully incorporated in human practices that normally presuppose moral agency.

9 For instance, see the disagreement between “functionalist” and “standard” views on moral agency described in Behdadi and Munthe (2020).

10 This echoes the “Integrative Social Robotics” (ISR) approach proposed by Seibt et al. (2018): “Currently social robotics and HRI research investigate what social robots *can* do, while robo-ethicists deliberate afterwards what robots *should* do. In contrast, according to the ISR approach we should ask what social robotics applications *can and should* do, from the very beginning” (p. 29).

11 For instance, Van Wynsberghe and Robbins (2019) argue that there are no good reasons to justify the creation of AMAs, and consequently, they propose a moratorium on “the commercialization of robots claiming to have ethical reasoning skills” (p. 732). In a similar vein, Metzinger (2021) has called for a global moratorium on synthetic phenomenology (i.e., artificial consciousness), as it could potentially cause an explosion of “negative valenced states” that would dramatically increase the amount of suffering in the universe.

12 Even more troubling is *scientific imperialism*, i.e., the phenomenon where one discipline aims to replace another discipline with its own methods (Mäki, 2013).

13 It is no coincidence that many emerging interdisciplinary fields have the term “computational” before the name of the traditional academic discipline it seeks to elucidate, including physics, chemistry, biology, sociology, law, and linguistics.

14 This mirrors the well-known conflict between, on the one hand empiricist, quantitative and causal methods, and on the other hand, anti-empiricist, qualitative and hermeneutic methods, that has divided disciplines in social science (Little, 1995).

15 Investment in AI continue to increase for every year, and in 2020, private investment in AI outnumbered public offerings by a factor of ten (Zhang et al., 2021).

16 “Ethics washing” occurs when ethics are instrumentally used as a deliberate communication strategy, occasionally as a of cover-up for unethical behavior (Bietti, 2020).

17 As Bourget and Chalmers (2014) concludes in their survey of the philosophical views of professional philosophers “There is famously no consensus on the answers to most major philosophical questions” (p. 492).

18 To be clear, this is not to say that philosophers in general—especially in light of the linguistic turn of twentieth century analytical philosophy—do not care about formal definitions. In fact, many philosophical projects are driven by the “inverse problem” of trying to find more precise and accurate definitions of important concepts and terms. More historically, one cannot deny the fundamental importance formal logic— from Aristotle’s *Organon* to Boolean algebra—had for philosophical analysis as well as for the development of computational systems (Gabbay & Woods, 2004). The point is rather that many philosophical inquires, in particular pertaining the moral realm, cannot be meaningfully construed or translated into a computational setting.

19 Although not possible to discuss within the scope of this paper, there are also combinations of EME and PME that can create potent forms of disciplinary synergy. In particular, philosophical views that assume that there are definable and quantifiable moral goods (e.g., certain forms of consequentialism) find strong synergies with AI methods that are based on the maximization of cumulative rewards (e.g., reinforcement learning).

20 Note that this might seem like a contradictory use of Kuhn’s notion of disciplinary matrix (and the closely related ‘paradigm’), since it is often used to denote the shared commitments a discipline needs in order to prosper in *normal science* (i.e., research within an established paradigm), and how it, in turn leads to incommensurability between paradigms (Kuhn, 1970). However, as Baalen and Boon (2019) argues, a disciplinary matrix provides part of the solution to the very problem it creates: by explicating the elements that constitute the matrix, researchers can acquire a meta-theoretical perspective on different matrices which in turn allows for cross- and interdisciplinary communication.

21 See Van Gulick (2018) for a comprehensive description of philosophical views on consciousness.

22 For instance, one might ask: how can machines ever tell right from wrong without the conscious experience of negative or positive mental states?

23 Intuitively, if someone had a possibility to do right yet acted wrongly, we hold them responsible for their wrongdoing..

24 I.e., while a self-driving vehicle is autonomous in the sense that it can drive from A to B without human control, it does not set its *own* goal, adhere to its *own* self-imposed rules (Kant, 2008), or have the choice to say “no” (Frankfurt, 1969).

25 See Sect. 4 in Tolmeijer et al. (2020).

26 See Sect. 5.3. in Tolmeijer et al. (2020) for specific evaluations used in machine implementations.

27 See also Cervantes et al. (2020) and Sect. 5 in Tolmeijer et al. (2020)

28 See Tolmeijer Sect. 7.3 for an exhaustive summary of these methods.

29 For instance, some authors argue that if there is small but non-zero chance that a catastrophic event *could* happen – e.g., existential catastrophe due to super-intelligent AI (Bostrom, 2017) or an explosion of artificial suffering (Metzinger, 2021) – it calls for serious academic and political consideration, even if we cannot properly assess the probability that the event occurs (epistemic uncertainty).
